# Drug susceptibility of *Plasmodium falciparum* in eastern Uganda: a longitudinal phenotypic and genotypic study

**DOI:** 10.1016/s2666-5247(21)00085-9

**Published:** 2021-06-18

**Authors:** Patrick K Tumwebaze, Thomas Katairo, Martin Okitwi, Oswald Byaruhanga, Stephen Orena, Victor Asua, Marvin Duvalsaint, Jennifer Legac, Sevil Chelebieva, Frida G Ceja, Stephanie A Rasmussen, Melissa D Conrad, Samuel L Nsobya, Ozkan Aydemir, Jeffrey A Bailey, Brett R Bayles, Philip J Rosenthal, Roland A Cooper

**Affiliations:** Infectious Diseases Research Collaboration, Kampala, Uganda; Infectious Diseases Research Collaboration, Kampala, Uganda; Infectious Diseases Research Collaboration, Kampala, Uganda; Infectious Diseases Research Collaboration, Kampala, Uganda; Infectious Diseases Research Collaboration, Kampala, Uganda; Infectious Diseases Research Collaboration, Kampala, Uganda; Department of Medicine, University of California, San Francisco, CA, USA; Department of Medicine, University of California, San Francisco, CA, USA; Department of Natural Sciences and Mathematics, Dominican University of California, San Rafael, CA, USA; Department of Natural Sciences and Mathematics, Dominican University of California, San Rafael, CA, USA; Department of Natural Sciences and Mathematics, Dominican University of California, San Rafael, CA, USA; Department of Medicine, University of California, San Francisco, CA, USA; Infectious Diseases Research Collaboration, Kampala, Uganda; Department of Pathology and Laboratory Medicine, Warren Alpert Medical School, Brown University, Providence, RI, USA; Department of Pathology and Laboratory Medicine, Warren Alpert Medical School, Brown University, Providence, RI, USA; Department of Natural Sciences and Mathematics, Dominican University of California, San Rafael, CA, USA; Department of Medicine, University of California, San Francisco, CA, USA; Department of Natural Sciences and Mathematics, Dominican University of California, San Rafael, CA, USA

## Abstract

**Background:**

Treatment and control of malaria depends on artemisinin-based combination therapies (ACTs) and is challenged by drug resistance, but thus far resistance to artemisinins and partner drugs has primarily occurred in southeast Asia. The aim of this study was to characterise antimalarial drug susceptibility of *Plasmodium falciparum* isolates from Tororo and Busia districts in Uganda.

**Methods:**

In this prospective longitudinal study, *P falciparum* isolates were collected from patients aged 6 months or older presenting at the Tororo District Hospital (Tororo district, a site with relatively low malaria incidence) or Masafu General Hospital (Busia district, a high-incidence site) in eastern Uganda with clinical symptoms of malaria, a positive Giemsa-stained blood film for *P falciparum*, and no signs of severe disease. Ex-vivo susceptibilities to ten antimalarial drugs were measured using a 72-h microplate growth inhibition assay with SYBR Green detection. Relevant *P falciparum* genetic polymorphisms were characterised by molecular methods. We compared results with those from earlier studies in this region and searched for associations between drug susceptibility and parasite genotypes.

**Findings:**

From June 10, 2016, to July 29, 2019, 361 *P falciparum* isolates were collected in the Busia district and 79 in the Tororo district from 440 participants. Of 440 total isolates, 392 (89%) successfully grew in culture and showed excellent drug susceptibility for chloroquine (median half-maximal inhibitory concentration [IC_50_] 20·0 nM [IQR 12·0–26·0]), monodesethylamodiaquine (7·1 nM [4·3–8·9]), pyronaridine (1·1 nM [0·7–2·3]), piperaquine (5·6 nM [3·3–8·6]), ferroquine (1·8 nM [1·5–3·3]), AQ-13 (24·0 nM [17·0–32·0]), lumefantrine (5·1 nM [3·2–7·7]), mefloquine (9·5 nM [6·6–13·0]), dihydroartemisinin (1·5 nM [1·0–2·0]), and atovaquone (0·3 nM [0·2–0·4]). Compared with results from our study in 2010–13, significant improvements in susceptibility were seen for chloroquine (median IC_50_ 288·0 nM [IQR 122·0–607·0]; p<0·0001), monodesethylamodiaquine (76·0 nM [44·0–137]; p<0·0001), and piperaquine (21·0 nM [7·6–43·0]; p<0·0001), a small but significant decrease in susceptibility was seen for lumefantrine (3·0 nM [1·1–7·6]; p<0·0001), and no change in susceptibility was seen with dihydroartemisinin (1·3 nM [0·8–2·5]; p=0·64). Chloroquine resistance (IC_50_>100 nM) was more common in isolates from the Tororo district (11 [15%] of 71), compared with those from the Busia district (12 [4%] of 320; p=0·0017). We showed significant increases between 2010–12 and 2016–19 in the prevalences of wild-type *P falciparum* multidrug resistance protein 1 (PfMDR1) Asn86Tyr from 60% (391 of 653) to 99% (418 of 422; p<0·0001), PfMDR1 Asp1246Tyr from 60% (390 of 650) to 90% (371 of 419; p<0·0001), and *P falciparum* chloroquine resistance transporter (PfCRT) Lys76Thr from 7% (44 of 675) to 87% (364 of 417; p<0·0001).

**Interpretation:**

Our results show marked changes in *P falciparum* drug susceptibility phenotypes and genotypes in Uganda during the past decade. These results suggest that additional changes will be seen over time and continued surveillance of susceptibility to key ACT components is warranted.

**Funding:**

National Institutes of Health and Medicines for Malaria Venture.

## Introduction

Malaria control in Africa has stalled in the past few years, and progress will be threatened by the emergence of resistance to the components of artemisinin-based combination therapies (ACTs), the first-line treatments for malaria caused by *Plasmodium falciparum*.^1,2^ ACT activity relies on a rapid-acting artemisinin derivative combined with a more slowly cleared partner drug. In Africa, artemether–lumefantrine and artesunate–amodiaquine are the first-line ACTs, and dihydroartemisinin–piperaquine, artesunate–mefloquine, and pyronaridine–artesunate are alternatives.^1,3^ For the prevention of seasonal malaria, amodiaquine plus sulfadoxine–pyrimethamine is recommended in parts of west and central Africa.^4^

Resistance to several antimalarials has spread to Africa after establishment in other regions.^2^ Clinical efficacies of the leading ACTs have remained excellent in African trials done in the past 5 years,^2-4^ but the emergence of resistance to artemisinins, ACT partner drugs, and chemoprevention regimens is a concern. Polymorphisms in the *P falciparum* chloroquine resistance transporter (PfCRT) and *P falciparum* multidrug resistance protein 1 (PfMDR1) are associated with resistance to chloroquine and amodiaquine.^5^ Until recently, most *P falciparum* parasites circulating in Uganda were chloroquine-resistant, and prevalence of the PfCRT Lys76Thr and PfMDR1 Asn86Tyr mutations was nearly 100%.^6,7^ These two mutations are also associated with resistance to amodiaquine, but generally not to the related compounds piperaquine or pyronaridine.^2^ Use of artesunate–amodiaquine selects for the Lys76Thr and Asn86Tyr substitutions in recurrent infections after therapy.^6^ These same substitutions are associated with increased susceptibility to lumefantrine and mefloquine, and the use of artemether–lumefantrine selects for wild-type loci in recurrent infections.^8,9^ In southeast Asia, but not convincingly in Africa, resistance to ACT partner drugs has been linked to amplification of *pfmdr1* for mefloquine and amplification of *plasmepsin 2–3* genes and novel PfCRT substitutions for piperaquine; these polymorphisms have been associated with resistance to artesunate–mefloquine and dihydroartemisinin–piperaquine.^2^ Definitive resistance to lumefantrine or pyronaridine has not been reported.^4^

*P falciparum* with decreased susceptibility to artemisinins, characterised by delayed clearance after treatment and the presence of kelch 13 (PfK13) propeller domain mutations, has emerged in the Greater Mekong subregion of southeast Asia.^10^ Polymorphisms in PfK13 occur at a low frequency in Africa, and those definitively associated with artemisinin resistance in Asia are particularly infrequent,^2^ although reports have identified PfK13 mutations that might mediate delayed clearance in Rwanda^11^ and Uganda.^12,13^

The *P falciparum* genetic landscape in Africa has changed as recommendations to treat uncomplicated malaria have progressed from chloroquine to ACTs (primarily artemether–lumefantrine in Uganda). In this context, we investigated the ex-vivo susceptibility to antimalarials of *P falciparum* isolates collected in 2016–19 from two sites in Uganda, resistance-associated genotypes of these isolates, and changes compared with our evaluations of isolates from the same region in 2010–13.

## Methods

### Source of isolates and sample collection

We did a longitudinal study of drug susceptibility phenotypes and genotypes from *P falciparum* causing malaria in eastern Uganda. *P falciparum* isolates were collected from patients aged 6 months or older presenting at the Tororo District Hospital (Tororo district) or Masafu General Hospital (Busia district) in eastern Uganda (appendix 1 p 4) with clinical symptoms of malaria, a positive Giemsa-stained blood film for *P falciparum*, and no signs of severe disease. Tororo District has low malaria transmission due to annual indoor residual spraying of insecticides,^14^ whereas the nearby Busia district does not use indoor residual spraying of insecticides and has high transmission. *P falciparum* is the dominant species in the region, and infections with non-falciparum malaria parasites are uncommon.^15^ Patients reporting use of antimalarial treatment within the previous 30 days or with evidence of an infection with other *Plasmodium* species were excluded. No other exclusion criteria were applied. Written informed consent was obtained from all participants. Parents or guardians of children younger than 18 years provided written consent on their behalf; children aged 8–17 years provided assent. 2–5 mL of venous blood was collected in a heparin tube by a laboratory technician before the start of therapy. Participants were given artemether–lumefantrine, following national guidelines, after sample collection. The study was approved by the Makerere University Research and Ethics Committee, the Uganda National Council for Science and Technology, and the University of California Committee on Human Research.

### Procedures

For parasite culture, parasitaemia was identified with Giemsa-stained thin films using a light microscope (CX21FS1; Olympus Corp, Tokyo, Japan) with a 100× objective lens and counting 1000 or more erythrocytes. Samples containing only *P falciparum* and a minimum of 0·3% parasitaemia were analysed. Due to logistical considerations (samples collected at different times of the day and travel time) isolates were stored at 4°C and assayed within 24 h of collection. Blood was centrifuged at 2000 revolutions per min for 10 min at room temperature, plasma and buffy coat were removed, and the erythrocyte pellet was washed three times with RPMI 1640 media (Thermo Fisher Scientific, Waltham, MA, USA) at 37°C. The pellet was resuspended in complete medium consisting of RPMI 1640 with 25 mM HEPES, 24 mM NaHCO_3_, 0·1 mM hypoxanthine, 10 μg/mL gentamicin, and 0·5% AlbuMAX II (Thermo Fisher Scientific, Waltham, MA, USA) to produce a haematocrit of 50%. The buffy coat was removed and four aliquots of approximately 10 μL were spotted onto Whatman 3MM filter paper (Cytivia, Marlborough, MA, USA) for molecular analysis.

To measure ex-vivo drug susceptibilities we used a 72 h microplate growth inhibition assay with SYBR Green detection, as previously described in the literature.^8^ Study compounds (chloroquine, AQ-13, monodesethylamodiaquine, ferroquine, piperaquine, pyronaridine, mefloquine, lumefantrine, dihydroartemisinin, and atovaquone), supplied by Medicines for Malaria Venture (Geneva, Switzerland), were dissolved in dimethyl sulfoxide (distilled water for chloroquine) as 10 mM stocks and stored at −20°C. Drugs were serially diluted by a factor of 3 in complete medium in 96-well microplates (50 μL per well), including drug-free and parasite-free control wells, with concentrations optimised to capture full dose–response curves (appendix 1 p 12; appendix 2 p 1). Cultures were diluted with uninfected O+ erythrocytes (from local blood banks) for total volumes of 200 μL per well at xrasitaemia and 2% haematocrit. Plates were maintained at 5% CO_2_, 5% O_2_, and 90% N_2_ for 72 h at 37°C in a humidified modular incubator (Billups Rothenberg, San Diego, CA, USA). After 72 h, wells were resuspended and 100 μL culture per well was transferred to black 96-well plates containing 100 μL per well SYBR Green lysis buffer (20 mM Tris, 5 mM EDTA (edetic acid), 0·008% saponin, 0·08% Triton X-100, and 0·2 μL/mL SYBR Green I [Invitrogen, Thermo Fisher Scientific, Waltham, MA, USA]), and mixed. Plates were incubated for 1 h in the dark at room temperature and fluorescence was measured with a FLUOstar Omega plate reader (BMG LabTech, Cary, NC, USA; 485 nm excitation and 530 nm emission). To monitor stability of drug stocks, laboratory control *P falciparum* Dd2 (MRA-156) and 3D7 (MRA-102) strains (BEI Resources, Manassas, VA, USA) were maintained in culture, synchronised with a magnetic column (Miltenyi Biotec, Auburn, CA, USA), and assayed (beginning at the ring-stage) once a month. Standard half-maximal inhibitory concentration (IC_50_) assays do not identify delayed clearance associated with dihydroartemisinin-resistant parasites in southeast Asia;^16^ therefore, susceptibility was measured using the ex-vivo ring-stage survival assay, which entails counting parasitaemias 66 h after a 6 h incubation with 700 nM dihydroartemisinin (appendix 1 p 2), as described elsewhere.^8,17^

For the genetic characterisation of PfCRT and PfMDR1, parasite DNA was extracted from filter paper blood spots using Chelex-100 (Bio-Rad, Hercules, CA, USA), relevant segments of the *pfcrt* and *pfmdr1* genes were amplified with nested PCR, and polymorphisms of interest were evaluated using a ligase detection reaction-fluorescent microsphere assay, as previously described (appendix 2 p 2).^18,19^

PfK13 sequences and copy number variations in *pfmdr1* and *plasmepsin 2–3* were analysed by molecular inversion probe (MIP) capture and deep sequencing.^20^ For MIP capture, DNA was isolated with Chelex-100 extraction buffer (0·5% Tween 20 instead of saponin). We designed a MIP panel with probes (appendix 2 p 3) targeting *pfk13*, *plasmepsin 2–3,* and *pfmdr1* using MIPTools software (version 0.19.12.13). MIP capture, library preparation, and sequencing were done as described in appendix 1 (p 2).^20^ Sequencing reads are available in the National Center for Biotechnology Information (NCBI) Sequence Read Archive (accession number PRJNA660547). Raw sequencing data were analysed using MIPTools (appendix 1 pp 2–3).^20^ Copy numbers were estimated on the basis of sample and probe normalised depth of sequence coverage from 31 unique probes for *pfmdr1* and 21 probes for the *plasmepsin 2–3* locus. The Dd2 strain, which contains amplified *pfmdr1* and is single copy for *plasmepsin 2–3*, was used as a control. For 29 samples collected, PfK13 sequences were analysed by dideoxy sequencing, and *pfmdr1* and *plasmepsin 2–3* copy numbers were analysed by quantitative PCR.^8^

### Statistical analysis

We assessed *P falciparum* drug susceptibility phenotypes and genotypes and compared results over time and between sites. Baseline characteristics of participants and isolates were computed as frequencies or means with SDs. Summary statistics for ex-vivo susceptibility of *P falciparum* isolates are median IC_50_ with IQR. Student’s *t* test was used to measure differences in mean parasitaemia, Mann-Whitney *U* test was used for median IC_50_ values, and Fisher’s exact test was used for proportions of resistant parasites between study sites. An IC_50_ value of 100 nM or higher defined chloroquine-resistant isolates, on the basis of studies indicating that this threshold is associated with clinical resistance.^21^ To quantify associations between ex-vivo drug susceptibilities, we calculated bivariate correlations between median IC_50_ values using Spearman’s rank-order correlation coefficient to account for non-parametric distributions of IC_50_ values. Changes in prevalence of genotypes was assessed using the χ^2^ test. To quantify temporal changes in drug susceptibilities, we used the Mann-Kendall non-parametric test to detect monotonic trends (change over time in a consistent positive or negative direction) with the R package Kendall. We calculated associations between transporter polymorphisms and ex-vivo susceptibilities by comparing IC_50_ values using the Mann-Whitney *U* test. All statistical tests were two-tailed, and results were considered statistically significant at a p value of less than 0·05. All statistical tests were done in R (version 3.4.4).

To assess well-to-well variability and signal-to-noise ratios in the fluorescence readout of ex-vivo assays, Z factors were calculated as: Z=1 − ([3×SD_infected drug free_ + 3 × SD_uninfected_] / [mean_infected drug free_ −mean_uninfected_]).^22^ IC_50_ were derived by plotting fluorescence intensity against log drug concentration and fit to a non-linear curve using a four-parameter Hill equation in Prism (version 9.0). For assays in which steep slopes resulted in a poor curve fit, slopes were fixed to a constant value of −6 (appendix 1 p 12). For results with incomplete curves at the lowest drug concentrations but at least 50% of the curve present, the upper plateau of the curve was constrained to the average growth in drug-free wells on the same assay plate.

### Role of the funding source

The funders of the study had no role in study design, data collection, data analysis, data interpretation, or writing of the report.

## Results

From June 10, 2016, to July 29, 2019, 361 *P falciparum* isolates were collected in the Busia district and 79 in the Tororo district, all from 440 patients with uncomplicated malaria. Baseline characteristics were similar between the sites (appendix 1 p 4). Mean parasitaemia of isolates was slightly greater in the Tororo district (3·9% [SD 2·9]) than in the Busia district (3·3% [2·6]; p=0·058), but this difference was not significant. Of 440 total isolates, 392 (89%) successfully grew in culture and 376 (85%) yielded ex-vivo susceptibility and genotyping data.

**Figure 1:**
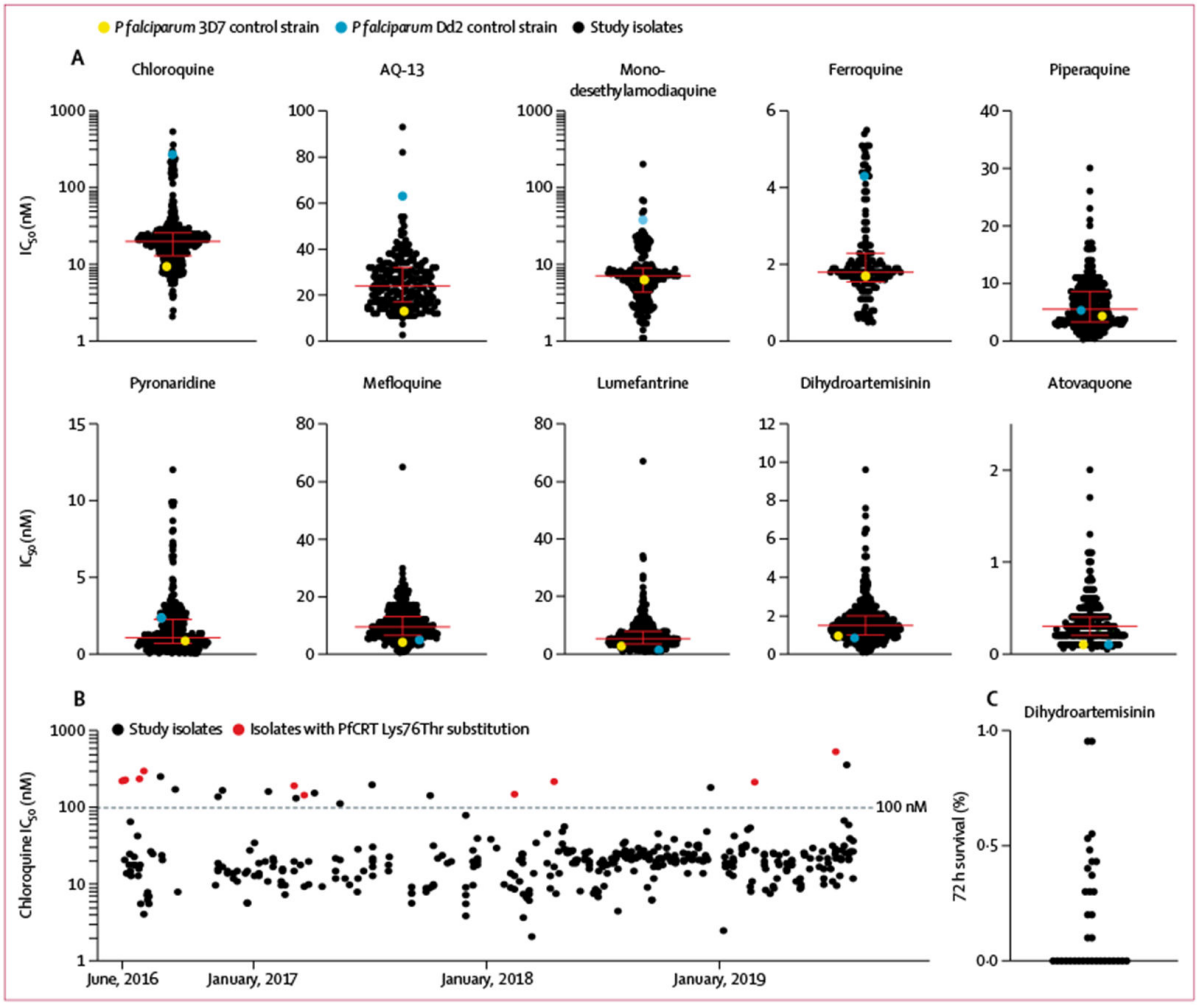
Ex-vivo susceptibility of *Plasmodium falciparum* isolates in 2016–19 Each point represents the result for a single isolate. (A) Median IC_50_ values and IQR are shown by the horizontal bars and whiskers. (B) Chloroquine susceptibility over time. Black dotted line shows a cutoff for chloroquine resistance (IC_50_≥100 nM). (C) Ex-vivo survival after exposure to dihydroartemisinin for 34 isolates. Parasite survival rates expressed as parasitaemia in dihydroartemisinin-pulsed cultures relative to parasitaemia in controls. Survival after dihydroartemisinin of less than 10% of that in the controls is considered a drug-sensitive response. IC_50_=half-maximal inhibitory concentrations.

We measured the ex-vivo susceptibility of up to 392 isolates to eight standard antimalarials and the experimental chloroquine analogues ferroquine^23^ and AQ-13^24^ (figure 1A; appendix 1 p 5). Mean annual Z factor ranged from 0·7 to 0·8, indicating robust, high-quality assays.^22^ Assessment of *P falciparum* Dd2 and 3D7 laboratory reference strains consistently yielded IC_50_ values similar to those reported previously (appendix 1 p 6).^8^ For field isolates, although drug resistance cutoffs are not defined for most antimalarials, median IC_50_ values for all tested drugs were at low nanomolar levels (less than 25 nM), consistent with potent activity (figure 1A). For chloroquine, susceptibility for 23 (5·9%) of 391 isolates exceeded the 100 nM threshold for resistance (figure 1B). For piperaquine, we did not observe biphasic dose–response curves, as seen for piperaquine-resistant field isolates from southeast Asia,^25^ even for the isolates with the highest IC_50_ values (exceeding 20 nM). For lumefantrine and mefloquine, only a single isolate for each drug was a marked outlier (outliers were not the same isolate). For dihydroartemisinin, the IC_50_ values were all low nanomolar; ex-vivo ring-stage survival assay of 16 isolates collected from June 13 to July 8, 2016,^8^ and 18 isolates collected from June 25 to July 26, 2019, showed that 18 (53%) of 34 had no parasites detectable after the 72 h incubation and 16 (47%) had parasitaemias of 0·1–1·0% of control values, consistent with an absence of the delayed clearance phenotype (figure 1C).

**Figure 2:**
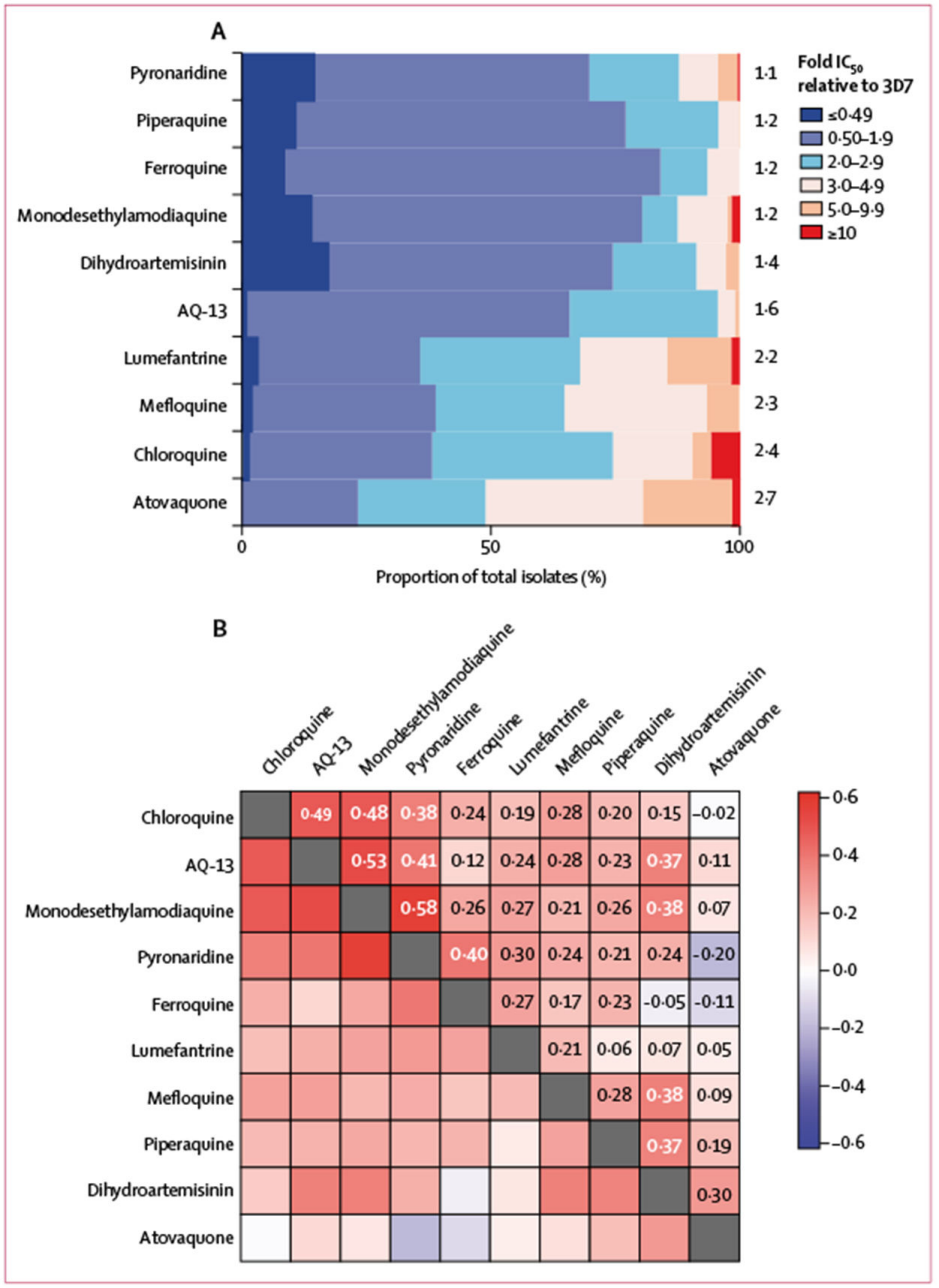
Analysis of ex-vivo susceptibility data (A) Susceptibility of Ugandan isolates in IC_50_ relative to the drug sensitive 3D7 control strain. The right y-axis labels indicate the mean fold increase in IC_50_ relative to 3D7 for each drug. (B) Correlations of ex-vivo susceptibilities between drugs. Magnitude and direction of associations between IC_50_ values are indicated by the colour and values. All correlation coefficients (≥0·15) were statistically significant (p<0·05; appendix 2 p 4). IC_50_=half-maximal inhibitory concentrations.

Most study isolates had similar susceptibilities to those in the drug-sensitive 3D7 control strain, with a small to moderate number of outliers (IC_50_ ≥5 times higher than that of 3D7) for pyronaridine (16 [4·3%] of 372), piperaquine (three [0·08%] of 376), monodesethylamodiaquine (six [2·5%] of 241), dihydroartemisinin (ten [2·7%] of 370), AQ-13 (three [1·3%] of 235), lumefantrine (53 [15·0%] of 365), mefloquine (27 [7·1%] of 378), chloroquine (38 [9·7%] of 391), and atovaquone (79 [22·0%] of 367; figure 2A).

Pairwise tests for correlations between ex-vivo drug susceptibilities offered potential insights into shared mechanisms, because compounds with IC_50_ values that correlated between isolates might share determinants of action or resistance (figure 2B). The strongest positive correlations (generally Spearman’s rank-order correlation coefficient ≥0·4) were observed between chloroquine, AQ-13, monodesethylamodiaquine, and pyronaridine, and between ferroquine and pyronaridine. All other associations were weak with notably weak correlations for results between piperaquine and all other aminoquinolines.

**Figure 3:**
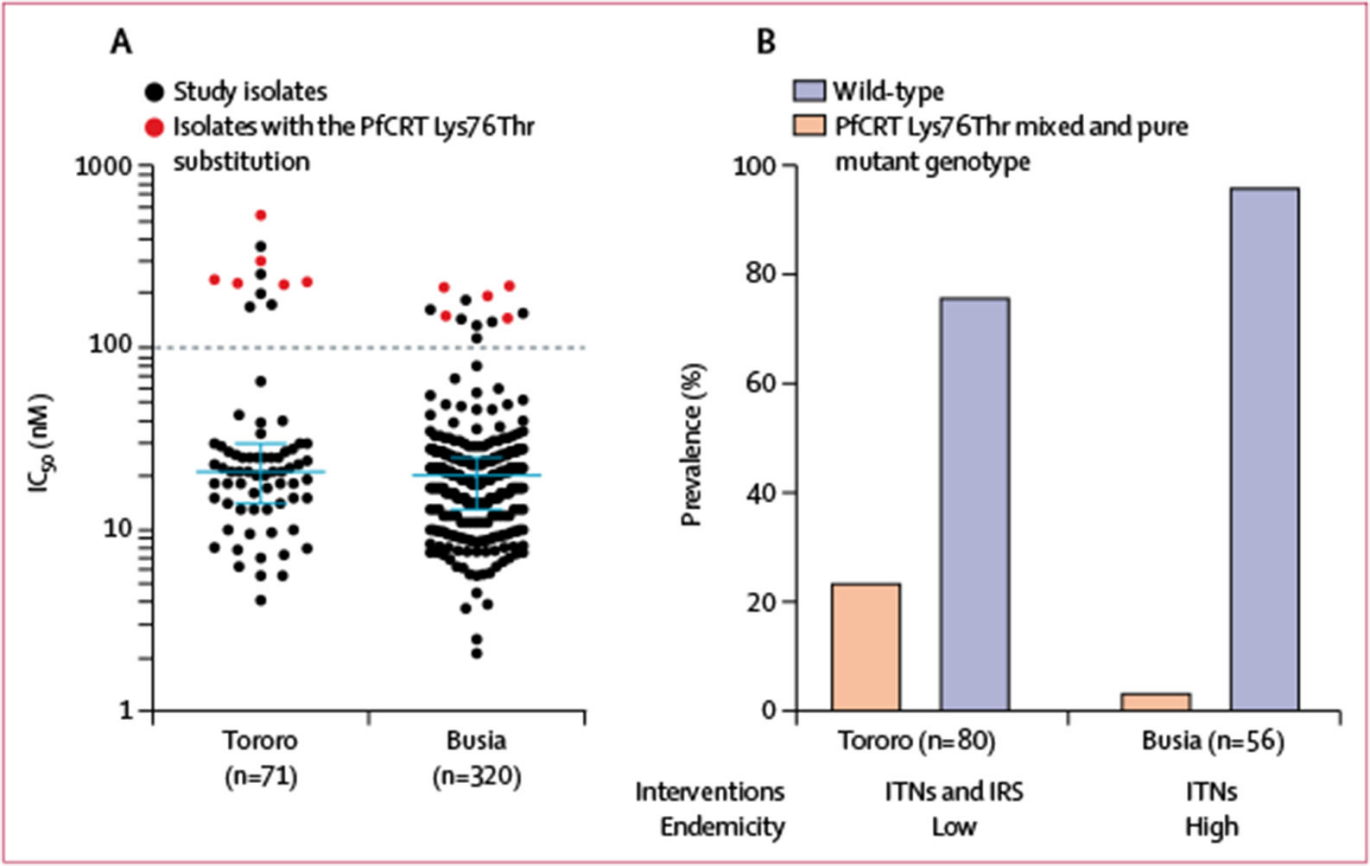
Chloroquine resistance in *Plasmodium falciparum* isolates in Tororo and Busia districts (A) Chloroquine susceptibility of isolates collected in 2016–19. Median IC_50_ values and IQR are shown by the blue bars and whiskers. Black dotted line shows a cutoff for chloroquine resistance (IC_50_ ≥100 nM). (B) Prevalence of isolates with the PfCRT Lys76Thr substitution (mixed and pure mutant genotypes) in 2018–19. IC_50_=half-maximal inhibitory concentrations. IRS=indoor residual spraying of insecticide. ITNs=insecticide-treated bednets. PfCRT=*P falciparum* chloroquine resistance transporter.

We compared drug susceptibilities and *pfcrt* genotypes between isolates collected in the Busia district, where malaria transmission is high, and Tororo district, where transmission is much lower due to annual indoor residual spraying of insecticides.^14^ Median susceptibilities of isolates from the two sites were similar (appendix 1 p 7). However, considering chloroquine resistant isolates (IC_50_>100 nM), resistance was seen in 11 (15%) of 71 isolates from the Tororo district versus 12 (4%) of 320 from the Busia district (p=0·0017; figure 3A), and the eight isolates with the highest IC_50_ values were all from the Tororo district. Considering genotypes, we compared isolates collected as part of routine surveillance from Nagongera (Tororo district) between May–June, 2018, and May–June, 2019,^13^ with those studied from the Busia district in the same time period, to control for potential temporal differences. We found significantly greater prevalence of the mutant PfCRT Lys76Thr chloroquine resistance mediator in Tororo (19 [24%] of 80) than in Busia (two [4%] of 56; p=0·0013; figure 3B). Thus, the high transmission site (Busia) had lower prevalence of chloroquine resistance than did the low transmission site (Tororo).

**Figure 4:**
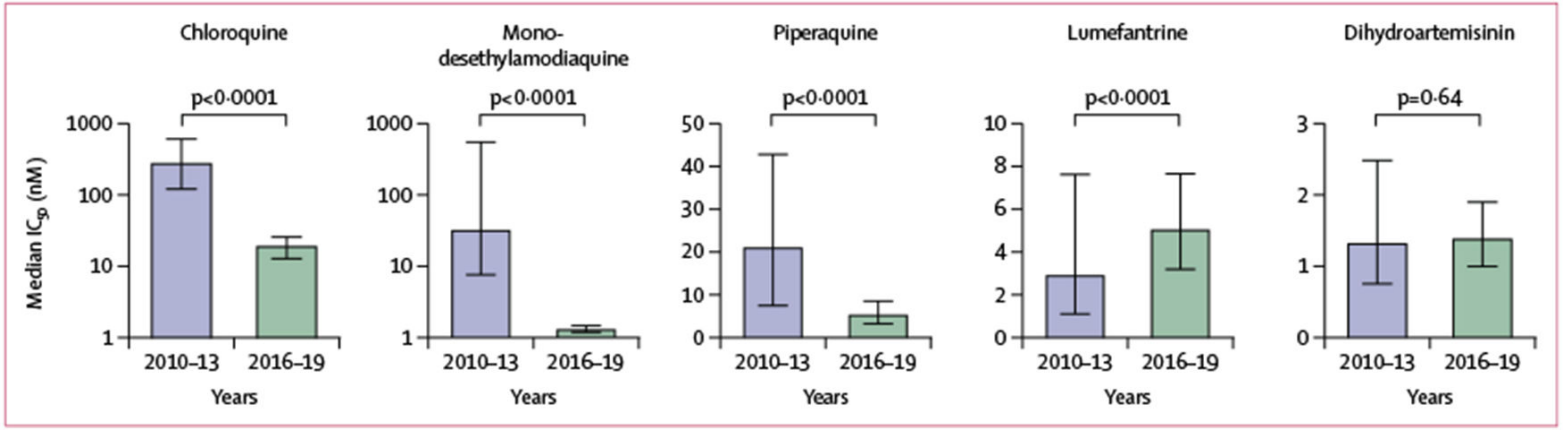
Ex-vivo drug susceptibility of Ugandan *Plasmodium falciparum* isolates over time Bars show IQR. Differences in IC_50_ values were calculated with the Mann-Whitney *U* test. IC_50_=half-maximal inhibitory concentrations.

Isolates we collected in 2016–19 showed excellent drug susceptibility for chloroquine (median IC_50_ 20·0 nM [IQR 12·0-26–0]), monodesethylamodiaquine (7·1 nM [4·3–8·9]), pyronaridine (1·1 nM [0·7–2·3]), piperaquine (5·6 nM [3·3–8·6]), ferroquine (1·8 nM [1·5–3·3]), AQ-13 (24·0 nM [17·0–32·0]), lumefantrine (5–1 nM [3·2–7·7]), mefloquine (9·5 nM [6·6–13·0]), dihydroartemisinin (1·5 nM [1·0–2·0]), and atovaquone (0·3 nM [0·2–0·4]).Compared with results from our study in 2010–13,^7^ significant improvements in susceptibility were seen for chloroquine (median IC_50_ 288·0 nM [IQR 122·0–607·0];p<0·0001), monodesethylamodiaquine (76·0 nM [44·0–137]; p<0·0001), and piperaquine (21·0 nM [7·6–43·0]; p<0·0001), a small but significant decrease in susceptibility was seen for lumefantrine (3·0 nM [1·1–7·6]; p<0·0001), and no change in susceptibility was seen with dihydroartemisinin (1·3 nM [0·8–2·5]; p=0·64; figure 4; several of the drugs were not part of the 2010–13 study). Considering chloroquine, the prevalence of ex-vivo resistance (IC_50_≥100 nM) decreased from 78% (317 of 408) in 2010–13 to 6% (23 of 391) in 2016–19. Clinically meaningful changes were not seen during this study (appendix 1 p 8). For chloroquine, some resistant isolates were identified each year, but their prevalence decreased over time, from 17% (nine of 52) in 2016 to 3% (three of 95) in 2019 (figure 1B).

**Figure 5:**
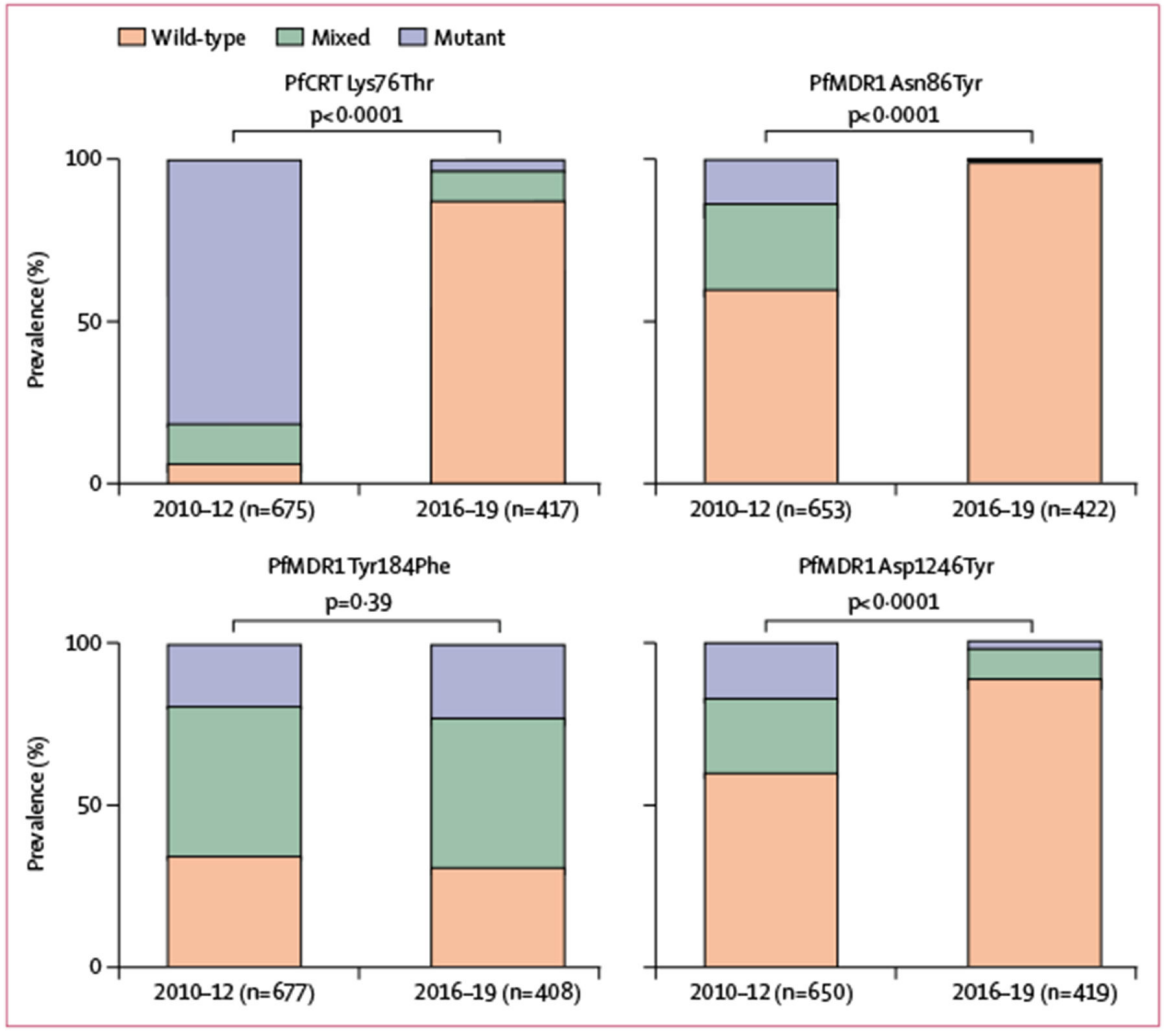
Prevalence of *Plasmodium falciparum* transporter substitutions over time p values for differences in the prevalence of wild-type versus mixed and pure mutant genotypes in 2010–12 and 2016–19 were calculated with a χ^2^ test. PfCRT=*P falciparum* chloroquine resistance transporter. PfMDR1=*P falciparum* multidrug resistance protein 1.

Changes in drug transporter genotypes—ie, substitutions in PfCRT and PfMDR1 associated with altered sensitivity to multiple antimalarials—were characterised for 424 isolates collected from 2016–19 (figure 5; appendix 1 p 9). For PfCRT Lys76Thr, the substitution most clearly linked to chloroquine resistance,^2^ isolates were predominantly wild-type (364 [87%] of 417) compared with isolates from 2010–12, when wild-type prevalence was low (44 [7%] of 675; p<0·0001). Similarly for PfMDR1, the prevalence of wild-type Asn86Tyr increased from 60% (391 of 653) to 99% (418 of 422; p<0·0001) and for Asp1246Tyr from 60% (390 of 650) to 90% (371 of 419; p<0·0001) between 2010–12 and 2016–19. The PfMDR1 Asn86Tyr substitution, associated with resistance to aminoquinolines, was detected in only four of 321 isolates from 2016–18 and none of 101 isolates from 2019. Prevalence of another highly polymorphic locus, PfMDR1 Tyr184Phe, which does not appear to be associated with drug sensitivity, was unchanged between 2010–12 and 2016–19.

We tested for associations between key *P falciparum* genetic transporter polymorphisms and ex-vivo drug susceptibilities (appendix 1 p 13). Assessments were limited by the low number of mutations at key loci, but nonetheless some important associations were seen. Notably, the PfCRT Lys76Thr substitution was associated with significantly decreased susceptibility to chloroquine, monodesethylamodiaquine, and AQ-13; associations with other aminoquinolines were not conclusive. Despite the availability of few mutant parasites for comparison, the PfMDR1 Asn86Tyr substitution appeared to be associated with increased susceptibility to lumefantrine and mefloquine, as seen previously.^8^

Amplification of genes associated with decreased susceptibility to mefloquine (*pfmdr1*) and piperaquine (*plasmepsin 2–3*) in southeast Asia^2^ was not detected in 126 isolates collected in 2016–18 and analysed by molecular inversion probe assays (appendix 1 p 14; appendix 2 p 3). Mean copy number estimates were 1·01 (range 0·92–1·20; n=126) for *pfmdr1,* 0·98 (0·66–1·33; n=126) for *plasmepsin 2*, and 0·98 (0·73–1·33; n=125) for *plasmepsin 3*. Considering isolates studied using different methods, amplification of *plasmepsin 2–3* was seen in four of 29 isolates collected in 2016 and analysed by qPCR,^8^ and no amplification of *pfmdr1* was observed in these 29 isolates.

Several PfK13 propeller domain mutations are associated with delayed parasite clearance after artemisinin therapy in southeast Asia.^2^ PfK13 sequences were available for 155 isolates. A few PfK13 propeller domain mutations were seen, mostly from isolates with mixed wild-type and mutant genotypes (appendix 1 p 10). Two substitutions previously associated with delayed clearance in southeast Asia^26,27^ and identified in samples from northern Uganda^12,13^ were seen: Cys469Tyr (three of 155) and Ala675Val (two of 149). Another substitution, Ala578Ser, which is the PfK13 propeller domain mutation most commonly reported in African isolates and does not mediate delayed clearance in transfected parasites,^2,28^ was seen in nine of 150 isolates.

## Discussion

Evaluation of ex-vivo drug susceptibility of malaria parasites is logistically challenging and can be done at only a few centres in Africa, yet whether the surveillance of molecular markers of resistance truly reflects drug susceptibility requires confirmation. We determined the ex-vivo drug susceptibilities of *P falciparum* isolates collected in eastern Uganda in 2016–19. We showed excellent susceptibility of isolates to all ten studied antimalarials. For chloroquine, for which resistance has gradually decreased in most of Africa probably because of reduced use of the drug in the past 20 years, 94% of isolates were highly susceptible, although some resistant parasites were observed. Ex-vivo susceptibility to the most important ACT partner drugs, such as lumefantrine, monodesethylamodiaquine, piperaquine, mefloquine, and pyronaridine, was generally excellent, and ring-stage survival assays did not show decreased susceptibility to dihydroartemisinin.

Molecular studies have measured the prevalence of known resistance markers, consistent with our ex-vivo susceptibility data and with recent trends, showing loss of markers of aminoquinoline resistance.^8,13^ Additionally, markers for artemisinin delayed clearance (PfK13 propeller domain mutations) and resistance to piperaquine (*plasmepsin 2–3* amplification or novel PfCRT mutations) or mefloquine (pfmdr1 amplification), all discovered in southeast Asia, were uncommon in Ugandan isolates, consistent with previous studies from Africa.^2^ Thus, reassuringly, *P falciparum* in Uganda is susceptible to components of the most important antimalarial drugs. However, *P falciparum* has changed remarkably in Uganda during the past decade, with increased susceptibility to some aminoquinolines and small decreases in susceptibility to lumefantrine.^8^ Additional changes, potentially mediating resistance to leading treatments, can be expected over time. Increasing prevalence of three K13 propeller domain substitutions—Cys469Tyr and Ala675Val in northern Uganda^12,13^ and Arg561His in Rwanda,^11^ which in Asia are associated with delayed clearance might threaten future efficacy of key ACTs.

Correlations between ex-vivo susceptibility results for different antimalarial compounds were informative because correlations in IC_50_ between compounds might highlight shared mechanisms of action or resistance. Notably, these assays were done with parasites generally susceptible to all studied compounds, so analyses pointed to shared mechanisms of action or resistance independent of high-level resistance. Results for the aminoquinolines chloroquine, monodesethylamodiaquine, and AQ-13 were highly correlated. However, correlations were not as strong with the related compounds pyronaridine, ferroquine, and piperaquine. Susceptibility to piperaquine appears to be mediated by alterations in drug transporters, as for related drugs, but different *pfcrt* mutations mediate resistance to chloroquine and piperaquine, consistent with an emerging understanding of structural determinants of drug transport.^29^

The current understanding of mediators of altered susceptibilities to antimalarial drugs is good.^2^ Our assessment of correlations between drug resistance genotypes and ex-vivo phenotypes supported previous analyses,^7,8^ although this study was limited by a small sample size for some previously common mutations. Overall, with marked decreases in prevalence of key *pfcrt* and *pfmdr1* mutations, parasites in Uganda are now generally highly susceptible to the ACT partners amodiaquine, piperaquine, and pyronaridine. Importantly, loss of these mutations is associated with decreased susceptibility to two other ACT partners, lumefantrine and mefloquine,^7,18,30^ but changes caused by reversion to wild-type at these loci are small and unlikely to have clinical consequences. However, if delayed artemisinin clearance and further decreases in lumefantrine susceptibility emerge in Uganda, the antimalarial efficacy of artemether–lumefantrine (first-line malaria treatment in the country) might be compromised, which remains a concern.

We studied the two investigational 4-aminoquinolines, ferroquine and AQ-13.^4,31^ Ferroquine, an analogue of chloroquine coupled with a ferrocene function, was highly potent against all studied Ugandan isolates, consistent with results from a 2019 study of isolates from Cambodia.^32^ AQ-13, a chloroquine derivative with a shorter diaminoalkane side-chain, had similar potency against Ugandan isolates as seen previously in culture-adapted *P falciparum* isolates from Tanzania,^33^ and showed promise in two small clinical studies, reviewed by Mengue and colleagues.^24^ Our results support ongoing consideration of these compounds as next-generation combination therapy partner drugs.

Our study included samples from two nearby sites that differ in one important regard. Tororo district previously had very high malaria transmission rates,^34^ but this transmission decreased markedly after introduction of annual indoor residual spraying of insecticides.^14^ The adjacent Busia district has similar ecology but did not receive indoor residual spraying of insecticides, and thus has persistently high transmission rates. Isolates from Tororo had a higher prevalence of ex-vivo chloroquine resistance and higher prevalence of the chloroquine resistance mediating PfCRT Lys76Thr substitution. This result is consistent with frequent use of artemether–lumefantrine in Busia but not Tororo, expediting selection of chloroquine-sensitive parasites.^7,18^ Although other factors might have contributed to differences between Busia and Tororo, and available data did not allow genotype–phenotype comparisons for all samples, this analysis suggests that local malaria control practices can have important effects on drug sensitivity, and that attention to local epidemiology is required to appropriately direct control efforts.

Our study had some limitations. First, ex-vivo drug susceptibility assays are inherently imprecise, and the results were most likely affected by varied growth in culture and the inability to repeat assays to improve precision. Second, to improve reliability, we limited our study to isolates with high parasitaemias; these results might not be representative of those for low density infections. Third, in areas with high malaria transmission, *P falciparum* infections are typically polyclonal, and our ex-vivo susceptibility values usually represented averages of results for competing clones in culture. Finally, our genotype–phenotype association studies were limited by decreasing prevalence of *P falciparum* genetic polymorphisms that previously mediated resistance to aminoquinolines.

In summary, our longitudinal ex-vivo studies showed that malaria parasites circulating in eastern Uganda are generally highly susceptible to artemisinins and ACT partner drugs. The gradual return of wild-type sequences at resistance-mediating loci in *pfcrt* and *pfmdr1* has been accompanied by a return of chloroquine susceptibility, but a slight loss in susceptibility to lumefantrine. Genetic and phenotypic markers for artemisinin and piperaquine resistance were uncommon, unlike in southeast Asia where resistance is a major problem. Our results are consistent with trials showing that leading ACTs remain efficacious for the treatment and prevention of malaria in Uganda.^35,36^ However, with an expectation that drug susceptibilities of circulating *P falciparum* parasites will continue to be affected by the selection pressures of commonly used drugs, maintaining surveillance is essential to detect changes in susceptibility to antimalarials.

## Supplementary Material

1

2

## Research in context

### Evidence before this study

We searched PubMed for the terms “antimalarial resistance”, “malaria”, “*Plasmodium*”, “Africa”, “ex vivo”, “pfmdr1”, “pfcrt”, “kelch”, or “K13” and identified papers published between Jan 1, 2000, and Dec 31, 2020, on antimalarial drug sensitivity and resistance in Africa. Additionally, we reviewed and included any relevant articles cited in those references. Our search identified many studies on antimalarial drugs and molecular markers of resistance, but few combining ex-vivo drug susceptibility with genotyping results.

### Added value of this study

This study focuses on expanding understanding of genotype–phenotype associations, supported by ex-vivo and genetic characterisations of antimalarial drug susceptibility and resistance. Our findings add value to the existing literature by providing comprehensive data on antimalarial drug susceptibility in Uganda during 2016–19. We assessed ex-vivo drug susceptibility of *Plasmodium falciparum*, evaluated changes in drug susceptibility over time, and characterised genotype–phenotype associations, considering genetic polymorphisms previously associated with resistance to various antimalarials.

### Implication of all the available evidence

Malaria parasites circulating in eastern Uganda in the past 5 years are mostly sensitive to commonly used antimalarial drugs. Furthermore, parasite genotypes and phenotypes have changed over time. There is no indication to change treatment recommendations for malaria in Uganda but, because continued changes can be expected over time, continued surveillance for antimalarial drug resistance is a high priority.
