# Supplementary material for: Drug susceptibility of Plasmodium falciparum in eastern Uganda: a longitudinal phenotypic and genotypic study
Source: Lancet Microbe. Author manuscript; Available in PMC 2021 Sep 21. (PMC8454895; doi:10.1016/s2666-5247(21)00085-9)
Supplement: 1 [file NIHMS1737664-supplement-1.pdf]

THE LANCET
Microbe

Supplementary appendix 1

This appendix formed part of the original submission and has been peer reviewed. We post it as supplied by the authors.

Supplement to: Tumwebaze PK, Katairo T, Okitwi M, et al. Drug susceptibility of *Plasmodium falciparum* in eastern Uganda: a longitudinal phenotypic and genotypic study. *Lancet Microbe* 2021; published online June 18. https://doi.org/10.1016/S2666-5247(21)00085-9.

SUPPLEMENTARY APPENDIX 1

Drug susceptibility of *Plasmodium falciparum* in eastern Uganda: a longitudinal phenotypic and genotypic study

Table of Contents

Supplementary Methods (pages 2 - 5)
Ex vivo ring-stage survival assay
MIP capture, amplification, and sequencing
MIP data processing and variant calling

Supplementary Tables (pages 6 - 12)
Supplementary Table S1: Baseline characteristics of patients and samples
Supplementary Table S2: Ex vivo drug sensitivity of *P. falciparum* isolates, 2016-19
Supplementary Table S3: Median IC_{50} values for *P. falciparum* laboratory control strains
Supplementary Table S4: Median ex vivo IC_{50} values for isolates summarized by collection location
Supplementary Table S5: Drug susceptibilities and temporal trends over time
Supplementary Table S6: PfCRT and PfMDR1 genotype prevalence over time
Supplementary Table S7: PfK13 mutations observed in isolates

Supplementary Figures (pages 13 - 16)
Supplementary Figure S1: Study sites in Tororo and Busia Districts in Eastern Uganda
Supplementary Figure S2: Representative dose-response curves from Ugandan *P. falciparum* isolates
Supplementary Figure S3: Associations between transporter polymorphisms and *ex vivo* susceptibilities
Supplementary Figure S4: Distribution of gene copy number estimates based on MIP genotyping for the *pfmdr1* and *plasmepsin 2* and *plasmepsin 3* genes

References (page 17)

Supplementary Appendix 2 (worksheets 1-4; Excel file: Appendix_Tumwebaze21.xlxs)
Drug dilutions for IC50s
Resources for PCR and LDR (ligase detection reaction)
MIP probe information
Spearman p values

Supplementary Methods

Ex vivo ring-stage survival assay. Parasite susceptibility to dihydroartemisinin (DHA) was assessed with the ex vivo ring-stage survival assay (RSA) as described.[1] Sixteen samples for RSA testing were collected from 13 June - 8 July 2016[2] and 18 samples from 5 June - 26 July 2019. Only parasite isolates that were ≥ 1% parasitemia were used for the RSA. After patient blood samples were collected and prepared for use in the ex vivo IC_{50} assay as described in Methods, cultures were adjusted to 2% hematocrit and 1% parasitemia with uninfected erythrocytes and complete media prewarmed to 37°C. Cultures were dispensed in 2 mL volumes to two separate wells in a sterile 24-well plate. One culture was treated with 700 nM DHA (in DMSO) and the other culture was adjusted to a final concentration of 0.1% DMSO. Plates were maintained under 5% CO_2, 5% O_2, and 90% N_2 for 6 h at 37°C in a humidified modular incubator until washing. At 6 h post-DHA exposure, cultures were transferred to a 15 mL conical centrifuge tube and washed 3 times by centrifugation (10 min @ 2000 rpm) with 10 mL of complete media containing 10 at 37 °C. Cultures were resuspended into unused wells in 2 mL volumes in complete media and cultured for an additional 66 h. Giemsa-stained thin smears were prepared from DHA- and DMSO-treated cultures 66 h post-wash (72 h after the start of the assay). Parasitemia was assessed in the DHA-treated culture by counting parasite-infected erythrocytes from 100 fields containing at least 100 total erythrocytes under 100X light microscopy (Model CX21FS1, Olympus Corp., Tokyo, Japan). An assay was considered successful if the control parasitemia was > 1.0%, indicating positive growth. Parasite survival rates were expressed as the parasitemia in the DHA-pulsed cultures relative to that in the DMSO controls, at the end of the 72 h assay. RSA percentages less than 10% are generally considered indicative of sensitivity to DHA.

MIP capture, amplification, and sequencing. Oligonucleotides described in Supplementary Appendix 2 were synthesized as 200 nmol ultramers (Integrated DNA Technologies, Coralville, IA, USA) with equimolar hand-mix option for random bases. These were pooled and 5' phosphorylated using 1 µl (10 units) T4 Polynucleotide Kinase (New England Biolabs, Ipswich, MA, USA) for every nanomole of probe, in 1X T4 DNA ligase buffer (New England Biolabs) in a maximum of 50 µl reaction (larger volumes were split). Phosphorylation reactions were incubated in a thermocycler at 37°C for 45 min followed by heat inactivation at 65°C for 20 min. Probes were aliquoted and kept at -20°C. Probes were diluted 1:8 in Tris-EDTA buffer to bring them to 1 µM working solution.

Capture reactions were carried out in 10 µl reactions for each sample consisting of Ampligase Buffer (1X; Lucigen Corp., Middleton, WI, USA), Phusion DNA polymerase (0.0008 units/µl; Thermo Fisher Scientific), Ampligase (0.04 units/µl; Lucigen Corp.), pooled MIPs (40 nM), dNTP (4 µM), and template DNA (5µl). These were incubated in a preheated thermocycler with the following steps: 95°C (10 min), 60°C (1 h), 4°C hold. Next, 2 µl of exonuclease mix, containing 1X Ampligase buffer, 10 units exonuclease I (New England Biolabs), and 50 units exonuclease III (New England Biolabs), were added to reactions and further incubated with the following steps: 37°C (1 h), 95°C (2 min), 4°C hold.

The entire capture reaction (12 µl) was used as template for a 25 µl PCR reaction containing 1X Phusion Polymerase Buffer (New England Biolabs), 1X Macromolecular Crowding (MMC) solution, 200 nM dNTP, 0.02 units/µl Phusion DNA polymerase (New England Biolabs), and 500 nM forward and reverse primers[3]. PCR was performed using a preheated thermocycler with the following steps: 98°C 30 s, 22 cycles (98°C 10 s, 63°C 30 s, 68°C 30 s), 68°C 2 min, 4°C hold. MMC stock (50 ml 5X) was prepared by mixing the following components in water and filter-sterilized using a 0.2 µ nylon syringe filter: 3.75 g Ficoll 70 (GE Healthcare, Chicago, IL, USA), 1.25 g Ficoll 400 (Sigma-Aldrich, St. Louis, MO, USA), 0.125 g polyvinylpyrrolidone (Sigma-Aldrich).

Next, library pools were created by combining 5 µl of each PCR reaction in a single tube and cleaned and concentrated using Ampure XP beads (Beckman Coulter, Brea, CA, USA) at a 0.8x bead:DNA ratio using the manufacturer's protocol. This generally removed smaller (~ 200 bp) unwanted adapter/primer dimers. If dimers remained after bead clean up, the eluted DNA was loaded on a 1.5% agarose gel, and the relevant band was extracted from the gel using a Monarch DNA extraction kit (New England Biolabs). Libraries were sequenced using dual indexing on an Illumina Nextseq 550 instrument (Illumina, San Diego, CA, USA) to generate 150 bp paired end sequences using Nextseq 500/550 Mid-output Kit v2.

MIP data processing and variant calling. Sequencing data were processed using MIPWrangler software (v1.1.1-dev, github.com/bailey-lab/MIPWrangler) in combination with other software. Briefly, sequences were demultiplexed by their dual sample barcode using bcl2fastq software (v2.20.0.422, Illumina). Paired end reads were

then stitched together using MIPWrangler and filtered on expected length and on per base quality scores by discarding a sequence if the fraction of quality scores above 30 was less than 70%. Quality filtered stitched sequences were then further demultiplexed by target using the extension and ligation arm sequences to produce a file for each target for each sample. Target sequences for each sample were then corrected using their unique molecular identifiers (UMIs). This was done by clustering sequences on their UMIs and then creating a consensus sequence for each specific UMI. This UMI redundancy removes a significant proportion of PCR errors that occur in late cycles, including polymerase stutter and subsequent sequencing errors. UMI corrected sequences were then further clustered within MIPWrangler using an implementation of the qluster algorithm derived from SeekDeep,[4] allowing accurate detection of single base differences and indels at levels of 1% or less. We set a minimum relative abundance threshold of 0.5% for a cluster to be included in the final analysis. Variant calls were carried out using freebayes (v1.3.1)[5] following alignment to the reference genome with bwa (v0.7.17),[6] and outputs were annotated using snpEff software (v4.3t).[7] Variants were filtered using MIPTools (v0.19.12.13) such that the variant site for a given sample had at least 5 UMI coverage and the non-reference allele had at least 2 UMIs supporting the call.

Copy numbers of *pfmdr1* and *plasmepsin 2/3* were estimated based on average depth of coverage for these genes using the cnv_caller module of MIPTools software. A basic algorithm was used to normalize the UMI count data: 1) filter samples with sufficient UMI coverage for a given gene (minimum 25 UMI per probe on average), 2) sample normalize UMI counts based on the mean of all MIPs in the sample to make samples with different total coverage comparable, 3) remove specific MIPs with high variability, 4) normalize the depth of each MIP across samples to the mean. The average value of all MIPs for a given gene and sample estimates the copy number of that gene for that sample. Average values were rounded to the nearest integer for discrete copy number calls.

Table S1. Baseline characteristics of patients and samples

	Busia Isolates (N = 361)		Tororo Isolates (N = 79)	
**Year of collection**	**Number samples**	**%**	**Number samples**	**%**
2016	22	6	38	48
2017	91	25	9	11
2018	161	45	17	22
2019	87	24	15	19
**Sex**				
Male	174	48	35	44
Female	187	52	44	56
**Age, years**				
< 5	248	69	60	76
≥ 5	113	31	19	24

Table S2. Ex vivo drug sensitivity (nM) of *P. falciparum* isolates, 2016-19

Drug	No. of isolates	Median IC_{50}	IQR
Chloroquine	391	20	12-26
AQ-13	235	24	17-32
Monodesethylamodiaquine	241	7.1	4.3-8.9
Ferroquine	139	1.8	1.5-3.3
Piperaquine	376	5.6	3.3-8.6
Pyronaridine	372	1.1	0.7-2.3
Mefloquine	378	9.5	6.6-13
Lumefantrine	365	5.1	3.2-7.7
Dihydroartemisinin	370	1.5	1.0-2.0
Atovaquone	368	0.3	0.2-0.4

IQR, interquartile range

Table S3. Median IC_{50} values (nM) for *P. falciparum* laboratory control strains

	3D7			Dd2		
**Drug**	**N**	**IC_{50}**	**IQR**	**N**	**IC_{50}**	**IQR**
Chloroquine	28	9.4	5.6 - 13	29	272	174 - 441
AQ-13	14	13	10 - 24	14	63	40 - 87
MDAQ	15	6.3	2.3 - 11	14	38	21 - 60
Ferroquine	14	1.7	1.4 - 1.8	13	4.3	2.5 - 5.1
Piperaquine	28	4.1	2.9 - 6.2	27	5.0	3.6 - 8.9
Pyronaridine	28	0.9	0.5 - 1.9	29	2.4	1.1 - 3.3
Mefloquine	28	4.1	2.5 - 5.8	29	4.9	1.8 - 7.3
Lumefantrine	27	2.5	1.7 - 3.1	27	1.1	0.7 - 1.9
DHA	28	0.9	0.6 - 1.5	28	1.0	0.7 - 1.2
Atovaquone	27	0.1	0.1 - 0.2	29	0.1	0.1 - 0.2

IQR, Interquartile range

Table S4. Median ex vivo IC_{50} values (nM) for isolates summarized by collection location

	Masafu Hospital, Busia District			Tororo District Hospital, Tororo District			
**Drug**	**N**	**IC_{50}**	**IQR**	**N**	**IC_{50}**	**IQR**	**p-value[a]**
Chloroquine	320	20	13 - 25	71	21	14 - 30	0.066
AQ-13	209	24	17 - 32	26	29	19 - 37	0.10
MDAQ	215	7.1	4.3 - 8.9	26	7.8	4.9 - 10	0.48
Ferroquine	118	1.8	1.5 - 2.2	21	1.9	1.6 - 2.5	0.38
Piperaquine	315	5.6	3.3 - 8.8	61	5.0	3.2 - 7.9	0.32
Pyronaridine	311	1.1	0.7 - 2.3	61	1.2	0.8 - 2.6	0.38
Mefloquine	316	9.3	6.4 - 13	62	11	6.8 - 22	0.26
Lumefantrine	302	5.1	3.1 - 7.8	63	5.1	3.9 - 7.6	0.78
DHA	309	1.5	1.0 - 2.0	61	1.4	1.1 - 1.8	0.90
Atovaquone	307	0.3	0.2 - 0.4	60	0.2	0.1 - 0.3	0.014

IQR, Interquartile range.

[a]Comparison of IC_{50} values between sites (Mann-Whitney U test).

Table S5. Drug susceptibilities (IC_{50}; nM) and temporal trends over time

Drug	Year	N	Median IC_{50}	IQR	Mann-Kendall tau[a]	p-value[b]
Chloroquine	2016	52	16	11 - 27		
	2017	86	17	9.9 - 22		
	2018	158	21	17 - 26		
	2019	95	21	16 - 27	0.12	0.00063
AQ-13	2016	-	-	-		
	2017	-	-	-		
	2018	141	24	17 - 31		
	2019	94	26	16 - 34	0.055	0.21
MDAQ	2016	-	-	-		
	2017	-	-	-		
	2018	147	7.3	4.9 - 9.3		
	2019	94	6.7	3.6 - 8.3	-0.0016	0.11
Ferroquine	2016	-	-	-		
	2017	73	1.8	1.4 - 2.0		
	2018	34	2.3	1.8 - 3.8		
	2019	32	1.8	1.5 - 2.2	0.099	0.091
Piperaquine	2016	42	4.1	3.1 - 6.7		
	2017	85	6.1	3.4 - 9.0		
	2018	156	5.3	3.2 - 8.7		
	2019	93	6.0	3.6 - 9.1	0.12	0.00035
Pyronaridine	2016	44	1.1	0.8 - 1.9		
	2017	85	0.9	0.5 - 1.8		
	2018	148	1.5	0.9 - 2.7		
	2019	95	0.9	0.6 - 1.8	0.040	0.26
Mefloquine	2016	44	8.8	6.5 - 12		
	2017	84	8.8	6.1 - 13		
	2018	155	10	6.9 - 14		
	2019	95	9.1	6.6 - 13	0.060	0.085
Lumefantrine	2016	44	6.3	4.7 - 8.8		
	2017	84	5.6	3.2 - 8.6		
	2018	153	4.8	2.4 - 6.7		
	2019	84	5.3	3.9 - 7.3	-0.033	0.35
DHA	2016	44	1.3	1.0 - 1.5		
	2017	85	1.9	1.2 - 2.7		
	2018	147	1.6	1.1 - 2.0		
	2019	94	1.2	0.9 - 1.6	-0.047	0.18
Atovaquone	2016	43	0.3	0.2 - 0.5		
	2017	84	0.4	0.3 - 0.6		
	2018	146	0.2	0.1 - 0.4		
	2019	94	0.2	0.1 - 0.3	-0.16	<0.00001

[a]Magnitude and direction of trend over time.
[b]Significance of increase or decrease in susceptibility over time (Mann-Kendall test).

Table S6. PfCRT and PfMDR1 genotype prevalence over time

			Genotypes, % (N)		
**Polymorphism**	**Year(s)**	**No. of Samples**	**Wild Type**	**Mixed**	**Mutant**
PfCRT Lys76Thr	2010-12	675	6.5 (44)	12.5 (84)	81.0 (547)
	2016	57	75.4 (43)	12.3 (7)	12.3 (7)
	2017	88	81.8 (72)	15.9 (14)	2.3 (2)
	2018	171	94.2 (161)	4.0 (7)	1.8 (3)
	2019	101	87.1 (88)	10.9 (11)	2.0 (2)
	2016-19	417	87.2 (364)	9.4 (39)	3.4 (14)
PfMDR1 Asn86Tyr	2010-12	653	59.9 (391)	26.5 (173)	13.6 (89)
	2016	58	98.3 (57)	1.7 (1)	0.0 (0)
	2017	90	97.8 (88)	1.1 (1)	1.1 (1)
	2018	173	99.4 (172)	0.0 (0)	0.6 (1)
	2019	101	100 (101)	0.0 (0)	0.0 (0)
	2016-19	422	99 (418)	0.5 (2)	0.5 (2)
PfMDR1 Tyr184Phe	2010-12	677	34.4 (233)	46.4 (314)	19.2 (130)
	2016	57	40.4 (23)	40.4 (23)	19.2 (11)
	2017	80	26.2 (21)	50.0 (40)	23.8 (19)
	2018	170	22.9 (39)	55.9 (95)	21.2 (36)
	2019	101	41.6 (42)	30.7 (31)	26.7 (2)
	2016-19	408	30.9 (126)	46.3 (189)	24 (93)
PfMDR1 Asp1246Tyr	2010-12	650	60.0 (390)	22.9 (149)	17.1 (112)
	2016	58	77.6 (45)	15.5 (9)	6.9 (4)
	2017	91	87.9 (80)	9.9 (9)	2.2 (2)
	2018	169	92.3 (156)	5.3 (9)	2.4 (4)
	2019	101	89.0 (90)	9.9 (10)	0.1 (1)
	2016-19	419	88.6 (371)	8.8 (37)	2.6 (11)

Data for 2010-12 were previously published.[8]

Table S7. PfK13 mutations observed in isolates[a]

PfK13 Locus	Wild Type (N)	Mixed (N)	Mutant (N)
Cys469Tyr	152	3	0
Ala578Ser	141	8	1
Val666Leu	148	0	1
Ala675Val	147	1	1

[a]N = 155; lower totals are due to unsuccessful sequencing reactions for the different *pfk13* loci.

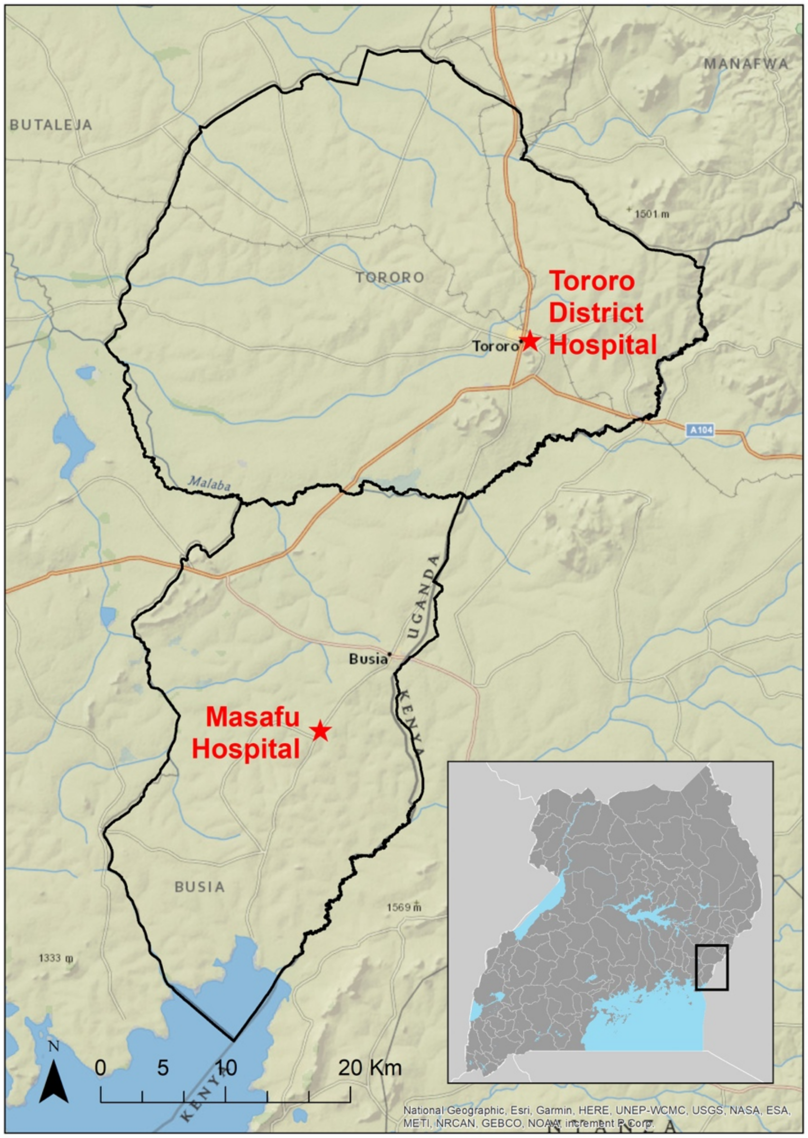

Figure S1. Study sites in Tororo and Busia Districts in Eastern Uganda.

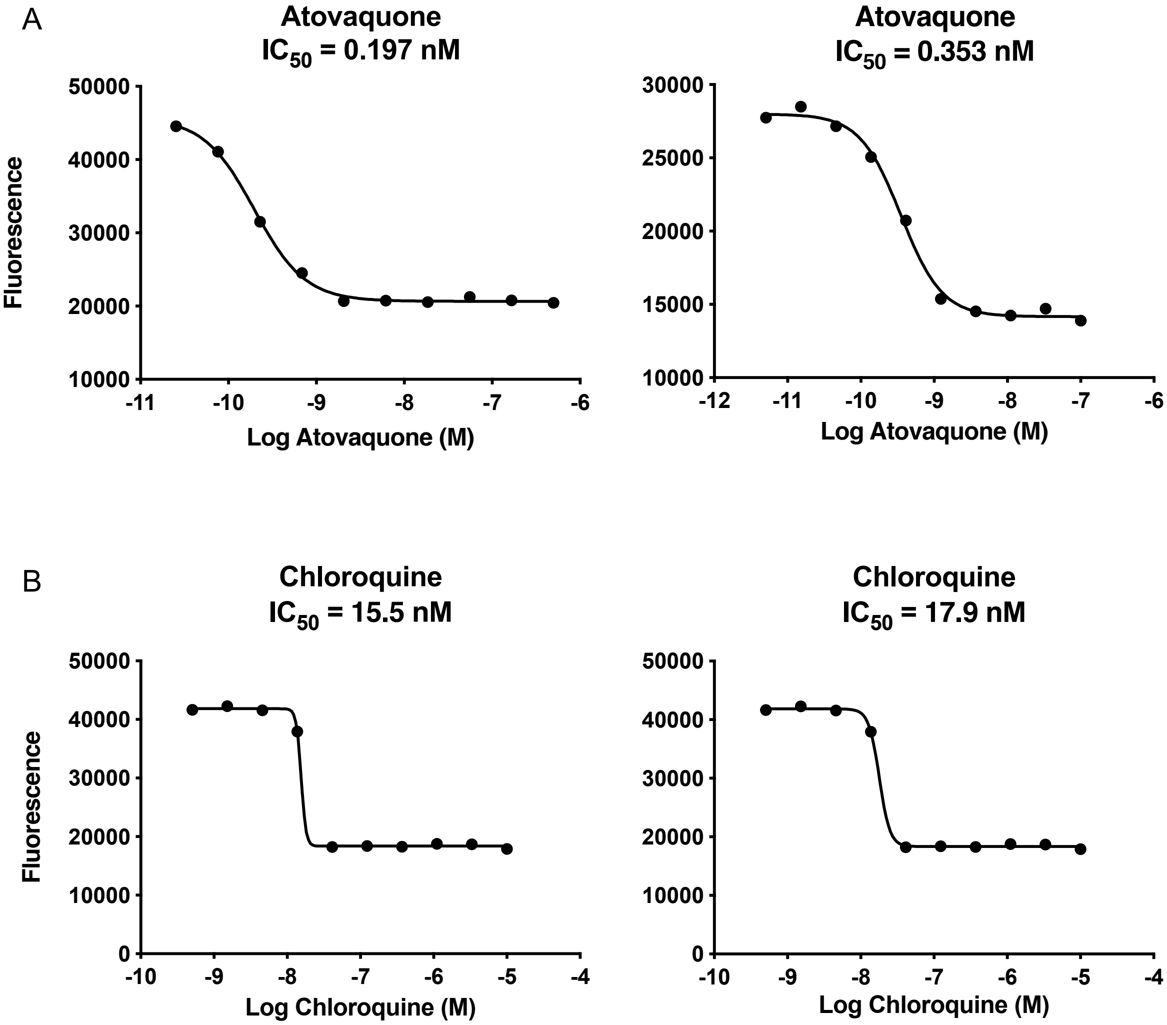

Figure S2. Representative dose-response curves from Ugandan *P. falciparum* isolates. (A) Graph on the left shows an assay resulting in an incomplete dose-response curve; that on the right shows an assay including lower drug concentrations that better captured the full curve. (B) Graph on the left depicts non-convergence of curve fit due to a steep slope and lack of data points in the steep portion of the curve; that on the right depicts the same assay results with the slope fixed to a constant of -6 to provide convergence of the curve fit.

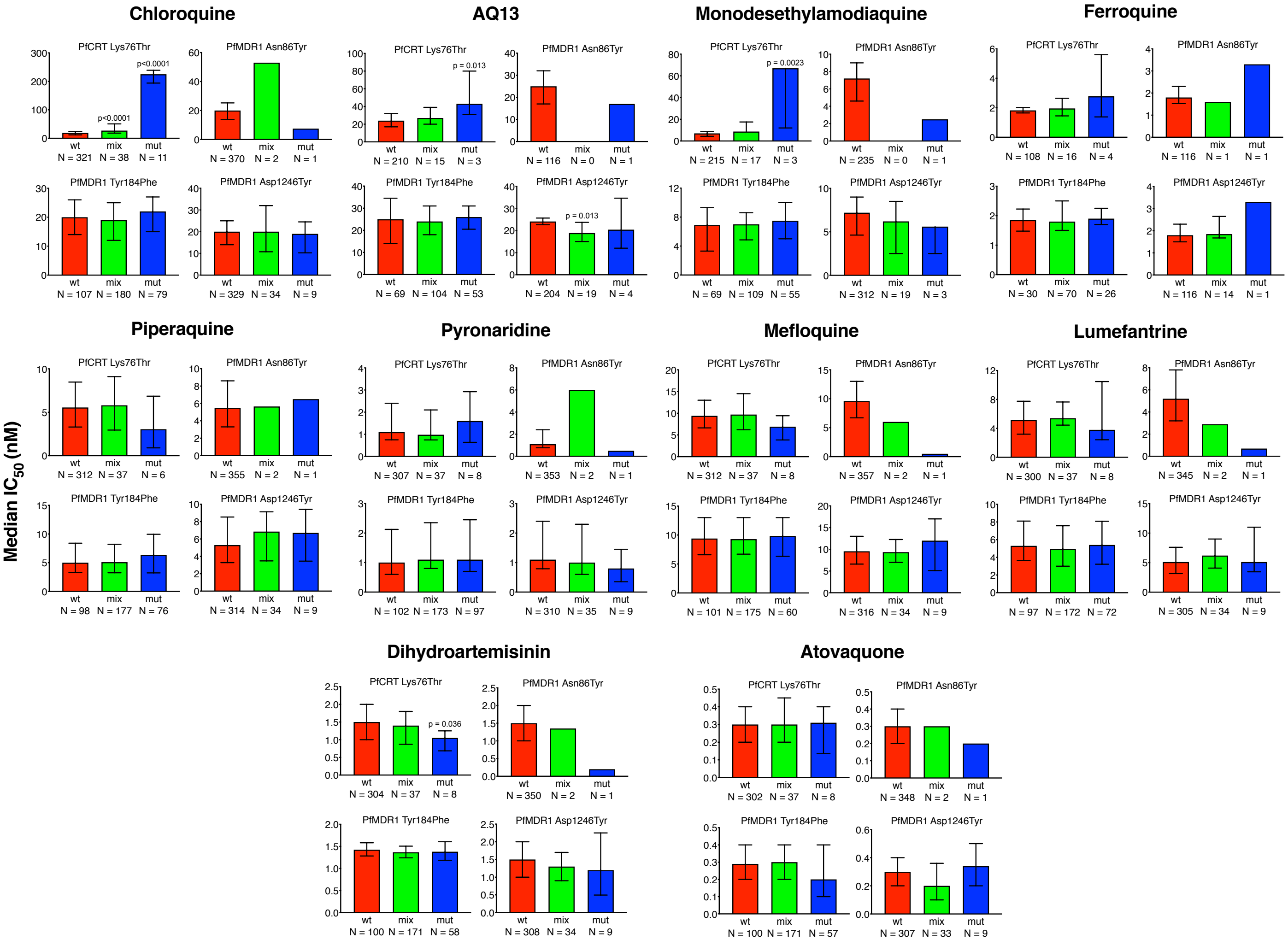

Figure S3. Associations between transporter polymorphisms and *ex vivo* susceptibilities. Bars represent median IC_{50} values with interquartile ranges (wt = wild type, mix = mixed wild type/mutant, mut = mutant). Interquartile ranges are not shown for $N \leq 2$, and these instances are not included in statistical tests. Statistically significant differences of IC_{50} values between wild type and other groups are indicated by p values (Mann-Whitney U test).

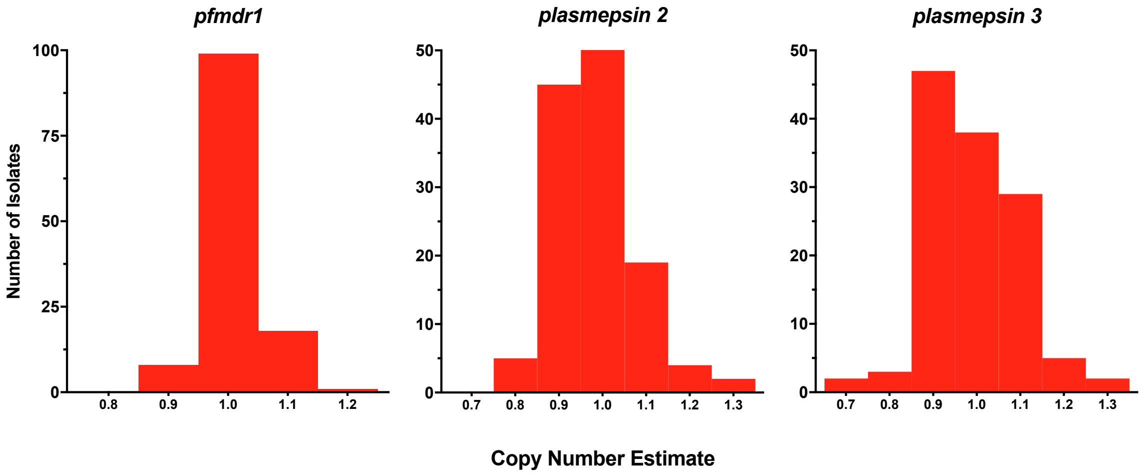

Figure S4. **Distribution of gene copy number estimates based on MIP genotyping for the *pfmdr1* and *plasmepsin 2* and *3* genes.** Calls are based on means from 31 unique probes for *pfmdr1* and 10 each for *plasmepsin 2* and *plasmepsin 3* (see appendix 2).

References

1. Witkowski B, Amaratunga C, Khim N, et al. Novel phenotypic assays for the detection of artemisinin-resistant *Plasmodium falciparum* malaria in Cambodia: in-vitro and ex-vivo drug-response studies. *Lancet Infect Dis* 2013; 13: 1043–9.
2. Rasmussen SA, Ceja FG, Conrad MD, et al. Changing antimalarial drug sensitivities in Uganda. *Antimicrob Agents Chemother* 2017; 61: e01516–17.
3. Aydemir O, Janko M, Hathaway NJ, et al. Drug-resistance and population structure of *Plasmodium falciparum* across the Democratic Republic of Congo using high-throughput molecular inversion probes. *J Infect Dis* 2018; 218: 946–55.
4. Hathaway NJ, Parobek CM, Juliano JJ, Bailey JA. SeekDeep: single-base resolution de novo clustering for amplicon deep sequencing. *Nucleic Acids Res* 2018; 46: e21.
5. Garrison E, Marth G. Haplotype-based variant detection from short-read sequencing. *arXiv* 2012; 1207.3907v2 [q-bioGN].
6. Li H. Aligning sequence reads, clone sequences and assembly contigs with BWA-MEM. *arXiv* 2013; 1303.3997v2 [q-bioGN].
7. Cingolani P, Platts A, Wang Le L, et al. A program for annotating and predicting the effects of single nucleotide polymorphisms, SnpEff: SNPs in the genome of *Drosophila melanogaster* strain w1118; iso-2; iso-3. Fly (Austin) 2012; 6: 80–92.
8. Tumwebaze P, Conrad MD, Walakira A, et al. Impact of antimalarial treatment and chemoprevention on the drug sensitivity of malaria parasites isolated from Ugandan children. *Antimicrob Agents Chemother* 2015; 59: 3018–30.
